# Time prediction model of subway transfer

**DOI:** 10.1186/s40064-016-1686-7

**Published:** 2016-01-19

**Authors:** Yuyang Zhou, Lin Yao, Yi Gong, Yanyan Chen

**Affiliations:** Beijing Key Laboratory of Traffic Engineering, Beijing University of Technology, Beijing, 100124 China

**Keywords:** Time prediction, Subway transfer, Passenger flow, Pedestrian facilities

## Abstract

Walking time prediction aims to deduce waiting time and travel time for passengers and provide a quantitative basis for the subway schedule management. This model is founded based on transfer passenger flow and type of pedestrian facilities. Chaoyangmen station in Beijing was taken as the learning set to obtain the relationship between transfer walking speed and passenger volume. The sectional passenger volume of different facilities was calculated related to the transfer passage classification. Model parameters were computed by curve fitting with respect to various pedestrian facilities. The testing set contained four transfer stations with large passenger volume. It is validated that the established model is effective and practical. The proposed model offers a real-time prediction method with good applicability. It can provide transfer scheme reference for passengers, meanwhile, improve the scheduling and management of the subway operation.

## Background

As an indispensable transportation system of big cities, subway has the merit of time reliability and vast capacity. With the development of urban subway system, passenger flow has increased significantly on peak time. The great concentration of passenger flow in transfer station has put enormous pressure on the subway system. Not only the underground network design decision, but also a huge range of consequential information service for passengers including transfer waiting time, travel time, reliability and even mode choice depend on the assumption about how long passengers will spent on walking from one subway line to the other.

It is shown from the literature about the subway interchange that Virkler and Elayadath (1994) established a relationship for speed-flow-density based on Transport and Traffic survey. Feng et al. (2009) drew the relation curve of speed and passenger flow density at the subway loading areas and upstairs in Beijing. Lu et al. (2002) founded the dynamical equation of evacuation speed for personnel. Fang et al. (2007) verified the dynamical equation of evacuation speed and the parameters in the equation. Kirchner and Schadschneder (2002) used a bionics-inspired cellular automaton model to analyze the pedestrian dynamics. Sarkar and Janardhan (1997) reported different speed relationships built for different pedestrian facilities. In the previous research, transfer time prediction was found region-specific. Bookbinder and Désilets (1992) developed its own emphasis on the evaluation of transfer time cost. Osorio and Bierlaire (2009) analyzed the capacity of the queuing network model which could capture the propagation of congestion and predict the travel time. Chen et al. (2008) analyzed the evacuation capacity and the level of the service for passengers at Metro station. Xuejun (2006) studied the characteristics of the transfer behavior from different transfer stations design. In the congestion situation, Singh and Srivastava (2008) proposed a traffic flow model which were applied to elevator configuration based on Markov network queuing theory. A new elevator-dispatching method was deduced theoretically using queuing theory by Zong et al. (2005). According to the survey on pedestrian walking characteristics, Blue and Adler (2001) established the cellular automata (CA) model based on the ant colony algorithms. Cao et al. (2009) put forward the update rules that could embody the walking characteristics of pedestrian counter flow.

Although the above researches cover most contents of the transfer behavior, there is less study for the walking time prediction by pedestrian facilities. Besides transfer speed characteristics, this paper analyzes the passenger walking behavior with respect to different pedestrian facilities in subway transfer stations. In “Transfer time prediction function” section, the time prediction model of the platform, the elevator and the horizon passage is established based on transfer flow volume and walking time on different facilities. In “Parameter estimation” section, model parameters are estimated according to the learning set. In “Transfer time model validation” section, the accuracy of transfer time model is validated by testing set including four transfer stations. Calculation results indicate that the transfer time prediction model can not only reduce waiting time for passengers, but also improve the subway operation efficiency.

## Transfer time prediction function

Transfer time *T* is considered as the time between passengers getting off one subway train and boarding another (Pepple and Adio 2014), which is divided into two parts: transfer walking time *T*_*w*_ and transfer waiting time *T*_*d*_. It is under the influence of transfer passenger flow and different facilities. Transfer walking time *T*_*w*_ includes the walking time on the platform *T*_1_, the time in the horizon passage *T*_2_ and that on the escalator *T*_3_.

1 $$T = T_{w} + T_{d} = T_{1} + T_{2} + T_{3} + T_{4}$$

Transfer walking time *T*_*i*_ on different facilities is calculated partly by:

2 $$T_{i} = \frac{{L_{i} }}{{v_{i} }}$$

For the facility *i*, *L*_*i*_ means the length, *v*_*i*_ means the speed.

When *i* = 1, 2, the section passenger flow *p*_*i*_ of transfer pedestrian facility *i* is:

3 $$p_{i} = \frac{N}{{B_{i} }}$$

where *N* means the number of transfer pedestrians, *B* means the width of different facilities.

Transfer speed on different facilities *v*_*i*_ is:

4 $$v_{i} = a_{i} p_{i}^{2} + b_{i} p_{i} + c_{i}$$

where *a*_*i*_, *b*_*i*_ and *c*_*i*_ are estimable parameters.

### Transfer walking on the platform

It is obvious that transfer walking time on the platform is related to the passenger distribution and the position of passages. Assume the transfer passenger distribution on the platform is uniform (Peng et al. 2009). Suppose *l*_1_ is the length of the platform, *D*(*x*) is the distance from a passenger’s position *x* to the position of the passage entrance, then the average length for transfer passengers walking to the passage is:

5 $$l_{1} = \int_{0}^{{l_{1} }} {\frac{D(x)}{{l_{1} }}} dx$$

### Transfer walking in the horizon passage

In the horizon passage, where is straight, long and non-stair, the main factor of walking speed is passenger volume. Pedestrians can walk freely when the transfer passenger volume is properly low (Canónico-González et al. 2013). However, in case the passengers get crowded, walking speed will decline gradually at peak time.

Walking on the stairs is also regulated with passenger volume while the speed is related to the climbing direction. According to the former research (Chen et al. 2011), the rate of upstairs walking time to horizon passage was 1.30 and the downstairs rate was 1.20. It should be noted that people would be more inclined to use the escalator instead of stairs.

### Transfer behavior on the escalator

As passengers on the escalator are restricted by the group behavior and the speed of the escalator, it is difficult for them to fascinate others. When transfer passengers overflow the escalator capacity, they have to pack together waiting for the queue in front of the escalator which will cause a sudden change of walking speed. In this case, the pedestrian arrivals distribution and the distribution of escalator service time are respectively assumed to be Poisson and negative exponential (Chen et al. 2012). Since the transfer station has more than one escalator, the queue system can be reduced M/M/S model (Li et al. 2014). Suppose *n* is the number of pedestrians in the queue system, *s* is the number of escalators. The pedestrian arrival rate is formulated as follows:

6 $$\lambda = v_{1} \cdot p_{1}$$

The service rate of the escalator is:

7 $$\mu = C_{1} \cdot B_{3} \cdot \omega$$

where *C*_1_ is the capacity of the escalator in units, *B*_3_ is the effected width of the escalator and *ω* is the saturation coefficient.

Based on the conclusion of the birth and death process, the stationary distribution in the queuing system is:

8 $$p_{n} = \frac{{\lambda^{n} }}{{\mu^{n} s!s^{n - s} }}p_{o}$$

*p*_0_ is the empty system probability, given by:

9 $$p_{0} = \sum\limits_{n = 0}^{s - 1} {\frac{{\rho^{n} }}{n!} + \frac{{\rho^{s} }}{{s!\left( {1 - \rho^{s} } \right)}}}$$

The average length of the queue is:

10 $$L_{q} = \sum\limits_{n = s + 1}^{\infty } {(n - s)p_{n} = \frac{{p_{0} \rho^{s} }}{s!}} \sum\limits_{n = s}^{\infty } {(n - s)} \rho_{s}^{n - s}$$

The average waiting time for the escalator is:

11 $$W_{q} = \frac{{L_{q} }}{\lambda }$$

Transfer time on the escalator is:

12 $$T_{3} = \left\{ {\begin{array}{*{20}l} {\frac{{L_{3} }}{{v_{t} }}} \hfill &\quad {\lambda \le \rho } \hfill \\ {W_{q} + \frac{{L_{3} }}{{v_{t} }}} \hfill & \quad {\lambda > \rho } \hfill \\ \end{array} } \right.$$

where *L*_3_ means the length of the escalator, *v*_*t*_ is the speed of the escalator.

### Waiting time

Another main factors affecting transfer time is the time passengers spend on waiting for the next train. Suppose one passenger gets off the subway at the time *t*_0_. When the passenger arrives at the receiving platform, the next schedule arrives at *t*_1_. The time one passenger spend in waiting is:

13 $$T_{d} = t_{1} - t_{0} - T_{w}$$

As can be seen from the above formula, the waiting time is influenced by the subway timetable and the time passengers spend in walking. If transfer walking time is equal to transfer time, the waiting time is reduced to zero. In such case, when a passenger arrives at the receiving platform, the subway is just about to leave.

## Parameter estimation

### Data acquisition

In Beijing, Chaoyangmen station is an important transfer station between subway Line 2 and Line 6 with 12,000 passengers per day. The research time lasted from 6:00 a.m. to 9:59 a.m. and from 4:00 p.m. to 7:59 p.m. The survey data set was divided into two parts. In the first subset, transfer volume was counted to analyze the sectional number of the transfer pedestrian. In the second one, walking time of transfer passengers was recorded to analyze the regulation of walking speed.

**Fig. 1:**
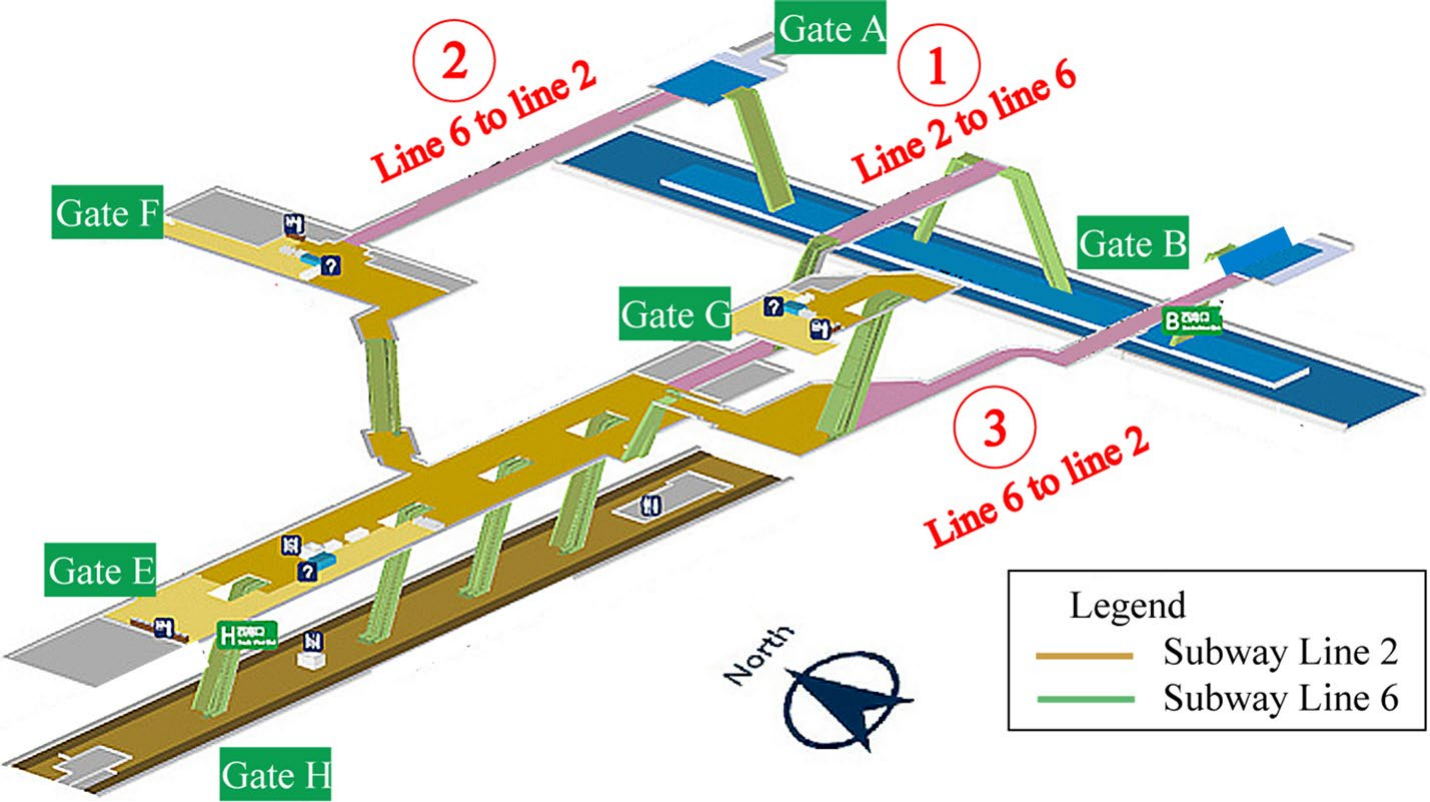
Transfer passages at Chaoyangmen station

The map of transfer passages at Chaoyangmen station is shown in Fig. 1. The passages are marked by the number 1, 2, and 3. Survey inspectors counted the number of passengers in each passage every 15 min. The survey got 96 records of transfer passenger volume including three passage, 8 h. The collected sample data of each passage was gathered into six cluster groups by the ranking of passenger volume.

**Fig. 2:**
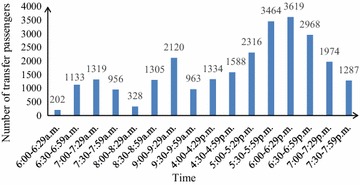
Transfer passenger volume from Line 2 to Line 6

**Fig. 3:**
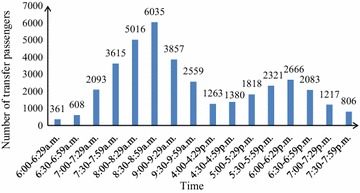
The transfer passenger volume from Line 6 to Line2

Figure 2 indicates that the number of transfer passengers from Line 2 to Line 6 is 26,876 in the survey and 46.10 % of that are counted during 5:00 p.m.–6:59 p.m. It’s found in Fig. 3 that the number of transfer passengers is 37,698 from Line 6 to Line 2, and 55.92 % of that are from 7:30 a.m. to 9:30 p.m. As transfer volume has significantly increased during the peak hour with reversibility, it is appropriate to select Chaoyangmen station for the survey.

The second subset aimed to investigate transfer time and the area size of different transfer facilities. Every quarter, the inspector selected a passenger at random and walking following through the transfer passage by the passenger’s choice. These survey data groups in different passages are shown in Table 1.

**Table 1:** The data groups in different passages

Passage no.	Group no.	*N* (p/min)	*v*_1_ (m/s)	*p*_1_ (p/m s)	*T*_1_ (s)	*v*_2_ (m/s)	*p*_2_ (p/m s)	*T*_2_ (s)	*v*_3_ (m/s)	*p*_3_ (p/m s)	*T*_3_ (s)	*T*_*w*_ (s)
1	1	11	1.73	0.02	4.91	1.25	0.05	85.60	0.50	0.15	50.04	140.55
	2	32	1.56	0.05	5.45	1.11	0.15	96.40	0.50	0.44	50.00	151.85
	3	45	1.46	0.07	5.82	1.05	0.21	101.90	0.50	0.63	50.10	157.83
	4	70	1.33	0.11	6.39	0.97	0.33	110.31	0.50	0.97	50.00	166.70
	5	121	1.27	0.19	6.69	0.88	0.58	121.59	0.41	1.68	69.26	197.54
	6	126	1.25	0.20	6.80	0.86	0.60	124.42	0.40	1.75	74.62	205.84
2	1	38	1.41	0.08	5.67	1.15	0.16	126.96	0.50	0.58	48.00	180.63
	2	42	1.39	0.09	5.76	1.12	0.20	130.36	0.50	0.64	47.86	184.07
	3	46	1.41	0.10	5.67	1.07	0.22	136.45	0.50	0.70	48.06	190.14
	4	71	1.26	0.15	6.35	1.00	0.34	146.00	0.50	1.08	48.10	200.35
	5	99	1.22	0.21	6.56	0.98	0.47	148.98	0.46	1.50	55.46	211.00
	6	119	1.17	0.25	6.84	0.95	0.57	153.68	0.38	1.80	66.54	227.06
3	1	7	1.75	0.01	4.57	1.31	0.03	103.05	0.50	0.11	47.92	155.54
	2	32	1.46	0.07	5.48	1.17	0.15	115.38	0.50	0.48	47.98	168.84
	3	44	1.42	0.09	5.63	1.15	0.21	117.39	0.50	0.67	48.06	171.09
	4	85	1.27	0.18	6.30	0.95	0.40	142.11	0.49	1.29	48.76	197.16
	5	129	1.17	0.27	6.84	0.93	0.61	145.16	0.36	1.95	72.50	224.50
	6	167	1.03	0.35	7.77	0.88	0.80	153.41	0.28	2.53	94.72	255.90

*p* number of transfer passengers, *v*_3_ means transfer speed on the escalator

The walking time spending on platform, escalator and horizon passage are recorded separately. The size of different areas and facilities were measured as below:

- Platform (Passage 1: effected width = 10.5 m and length of measurement section = 3.3 m; Passage 2: effected width = 8.0 m and length of measurement section = 3.5 m; Passage 3: effected width = 8.0 m and length of measurement section = 3.5 m);
- Escalator (Passage 1: effected width = 1.2 m and length of measurement section = 12.0 m; Passage 2: effected width = 1.1 m and length of measurement section = 12.0 m; Passage 3: effected width = 1.1 m and length of measurement section = 12.0 m; the speed of escalators: 0.5 m/s);
- Horizon passage (Passage 1: effected width = 3.5 m and length of measurement section = 107 m; Passage 2: effected width = 4.0 m and length of measurement section = 146.0 m; Passage 3: effected width = 4.0 m and length of measurement section = 135.0 m).

Assume *v*_1_, *v*_2_ and *v*_3_ come from the same population. The statistical software SPSS is applied to verify the independent sample with Mann–Whitney U method and Kolmogorov–Smirnov Z method. The test result is shown in Table 2.

**Table 2:** Results of Mann–Whitney and Kolmogorov–Smirnov tests

Mann–Whitney tests	*v*_*i*_
Mann–Whitney U	0.000
Wilcoxon W	4656.000
Z	−12.153
Significance (both tails)	0.000
Kolmogorov–Smirnov tests	
Extreme difference	
Absolute value	1.000
Positive	1.000
Negative	0.000
Kolmogorov–Smirnov Z	6.928
Significance (both tails)	0.000

*H*_0_: *v*_1_, *v*_2_ and *v*_3_ come from the same population.

*α* = 0.05.

In Table 2, results indicate that the both tails from Mann–Whitney U test and Kolmogorov–Smirnov *Z* are *p*_1_ = 0.000 < *α* and *p*_2_ = 0.000 < *α*. Thus, *H*_0_ is rejected. It means *v*_1_, *v*_2_ and *v*_3_ show significant differences. Therefore, to analyze the walking speed and transfer time with respect to various pedestrian facilities has necessity.

### Walking time model on the platform

Passengers tend to get impatient easily, since they get off from the crowded subway. As transfer volume increases, the sectional number of transfer pedestrians is getting higher. It leads to the decline of the walking speed. As shown in the Table 2, the speed obtained from the platform concentrates on 1.2–1.5 m/s, which is higher than usual. When the sectional number increases to 0.35 (p/m s), the average speed declines to 1.03 m/s. The formula (4) below is built by the fitting method in MATLAB 2013.

The collected data is fitted by the quadratic function. The formula for the speed and the sectional number from the platform is:

14 $$v_{1} = 5.427p_{1}^{2} - 3.72p_{1} + 1.729$$

The value *R*^2^ of significant degree of regress is 0.937, which indicates that *v*_1_ is highly related to *p*_1_ when *p*_1_ is from 0.01 to 0.35. Based on the position of the platform and the sectional number, the formula for the walking time and the sectional number from the platform is:

15 $$T_{1} = \frac{{L_{1} }}{{v_{1} }} = \frac{{\int_{0}^{{l_{1} }} {\frac{D(x)}{{l_{1} }}dx} }}{{5.427p_{1}^{2} - 3.721p_{1} + 1.729}}$$

### Walking time model in horizon passage

The data of the speed obtained from horizon passage concentrates on 0.9–1.2 m/s. Passengers in horizon passage have less space than that on the platform. Thus the speed in horizon passage is slower. Therefore, the sectional number in horizon passage is the main factor affects the walking time. Formula (4) is built by the fitting method in MATLAB 2013.

The collected data is fitted by the quadratic function. The formula for the speed and the sectional number from the platform is:

16 $$v_{2} = 1.023p_{2}^{2} - 1.572p_{2} + 1.732$$

The value *R*^2^ of significant degree of regress is 0.966, which shows that *v*_2_ is highly related to *p*_2_ when *p*_2_ is the range of 0.03–0.80. The formula for the walking time and the sectional number from the horizon passage is:

17 $$T_{2} = \frac{{L_{2} }}{{v_{2} }} = \frac{{L_{2} }}{{1.023p_{2}^{2} - 1.572p_{2} + 1.732}}$$

### Walking time model on the escalator

**Fig. 4:**
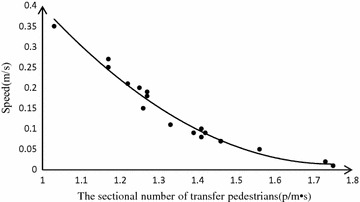
Relation between speed and the sectional number of platform

The speed of the transfer passengers is almost identical to the escalator speed when the number of people is under the capacity of the escalator. Passengers will not wait in line to get on the escalator until the sectional transfer number increases to 1.29 (p/m s). The capacity of the escalator is *A*_1_ = 8100 (p/m h) (Chen et al. 2008). The speed of the escalator is *v*_*T*_ = 0.5 m/s. Based on the collected data in Fig. 4, the saturation coefficient *ω* in the formula (7) is 0.092.

**Fig. 5:**
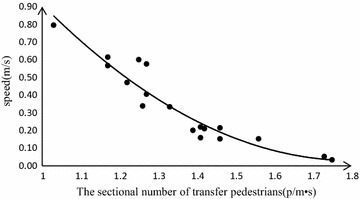
Relation between speed and density of horizon passage

Through the above analysis, the model parameters in formula (1) are defined step by step. In case the sectional number of transfer pedestrians is known, transfer walking time can be calculated through the model in Fig. 5.

## Transfer time model validation

**Fig. 6:**
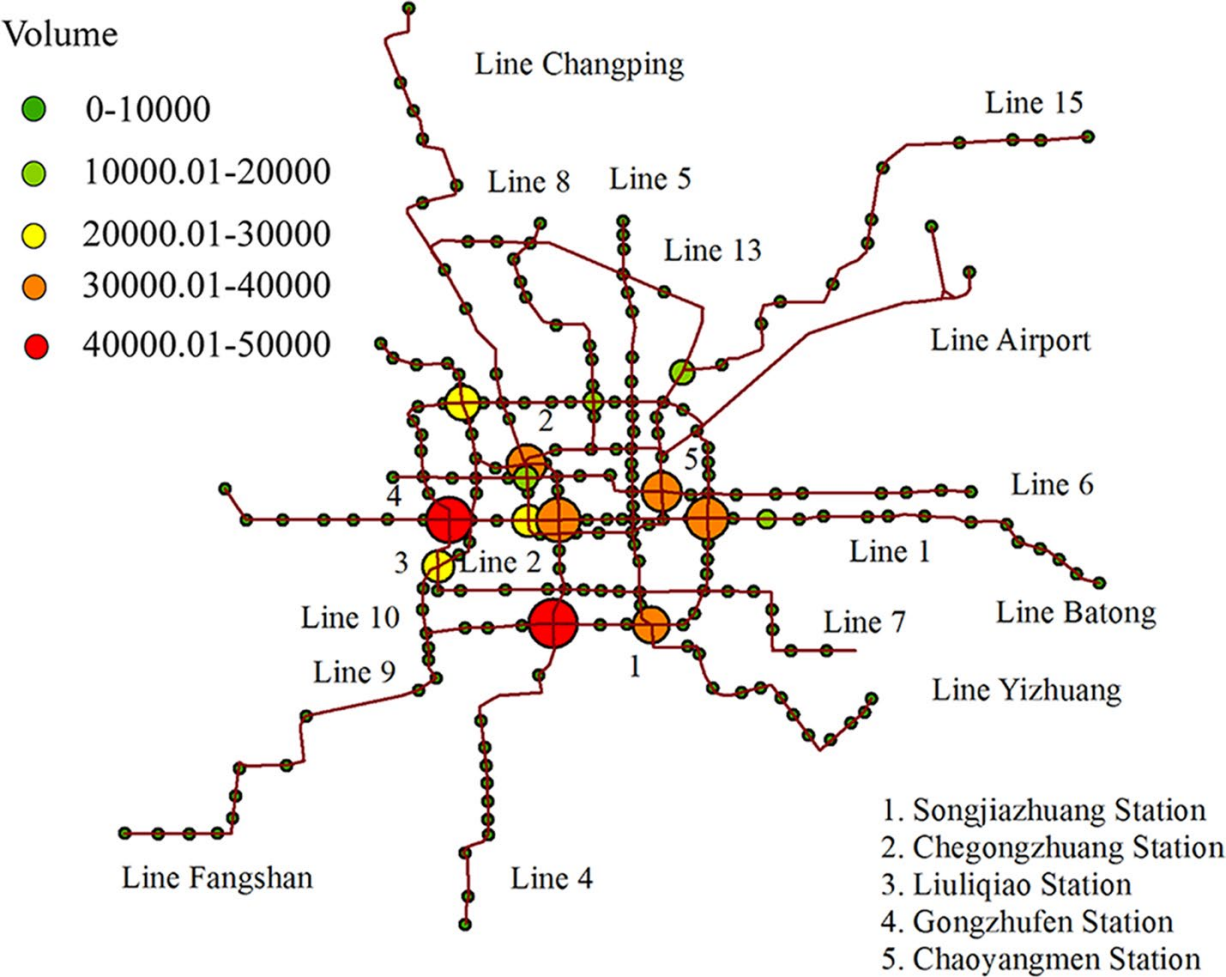
Beijing subway lines and transfer stations

Considering transfer volume and the affection of different facilities, four other transfer stations of different lines were selected to be the texting set. The data from Songjiazhuang station (Line 5, Line 10 and Line Yizhuang), Chegongzhuang station (Line 2 and Line 6), Liuliqiao station (Line 9, Line 10 and the coach station) and Gongzhufen station (Line 1 and Line 10) (Fig. 6) were collected every quarter during 3:00 p.m.–5:00 p.m. as off-peak time and from 5:00 p.m. to 7:00 p.m. as peak hours, on October 28th, 2014. The survey lasted for 4 h in these four stations connecting seven lines. The 64 sample data were clustered into 16 groups by the ranking of transfer time. Assuming the waiting time takes up half of the departure time interval, the survey data and calculates results through the texture model are given below by the transfer volume ranking using GIS (Li et al. 2014).

From Table 3, the measured time is counted in single digits which have a sampling error of ±1 s to the real time. The error rate of measured time is tiny compared to the real time within the range of 0.002–0.005. The average value and the standard deviation of the error rate for the texture model are 3.50 % and 0.025. As the results based on different transfer stations and subway lines, it is indicated that the proposal transfer time prediction model is stable and practical. Besides, the testing set which includes various transfer passages and different transfer volume illustrates that the model has good robustness.

**Table 3:** The calculation results in different stations

Group no.	*N* (p/min)	*L*_1_ (m)	*B*_1_ (m)	*v*_1_ (m/s)	*p*_1_ (p/m s)	*L*_2_ (m)	*B*_2_ (m)	*v*_2_ (m/s)	*p*_2_ (p/m s)
1	6	2.80	11.50	1.73	0.01	88.50	4.50	1.29	0.02
2	9	3.20	9.00	1.26	0.02	123.00	3.50	1.26	0.04
3	15	2.60	10.50	1.40	0.02	142.50	4.50	1.21	0.06
4	29	3.50	8.50	1.53	0.06	105.50	4.50	1.14	0.11
5	33	3.30	8.00	1.45	0.07	152.50	4.50	1.12	0.12
6	34	4.20	6.50	1.48	0.09	115.50	4.50	1.10	0.13
7	43	4.00	6.00	1.34	0.12	133.00	3.50	1.07	0.20
8	64	3.50	6.00	1.24	0.18	97.00	4.50	1.03	0.24
9	82	3.50	6.00	1.76	0.23	97.00	4.50	0.94	0.30
10	96	4.20	6.50	1.23	0.25	115.50	4.50	0.96	0.36
11	105	2.60	10.50	1.43	0.17	142.50	4.50	0.94	0.39
12	110	4.00	6.00	1.19	0.31	133.00	3.50	0.89	0.52
13	122	3.20	9.00	1.41	0.23	123.00	3.50	0.91	0.58
14	143	3.30	8.00	1.24	0.30	152.50	4.50	0.83	0.53
15	151	2.80	11.50	1.39	0.22	88.50	4.50	0.81	0.56
16	158	3.50	8.50	1.29	0.31	105.50	4.50	0.80	0.59

Group no.	*L*_3_ (m)	*B*_3_ (m)	*v*_3_ (m/s)	*p*_3_ (p/m s)	*T* (s)	*T*_*tr*_ (s)	Error rate (%)
1	24.00	1.20	0.50	0.08	190.76	191	0.13
2	28.00	1.10	0.50	0.14	334.73	343	2.41
3	25.00	1.20	0.50	0.21	307.56	307	0.18
4	25.00	1.10	0.60	0.44	228.10	231	1.26
5	25.00	1.20	0.60	0.46	316.25	333	5.03
6	25.00	1.10	0.50	0.52	249.65	241	3.59
7	28.00	1.10	0.60	0.65	348.78	339	2.88
8	24.00	1.20	0.50	0.89	214.93	224	4.05
9	24.00	1.20	0.49	1.14	223.37	236	5.35
10	25.00	1.10	0.46	1.45	270.50	255	6.08
11	25.00	1.20	0.41	1.46	351.75	359	2.02
12	28.00	1.10	0.38	1.67	397.62	408	2.54
13	28.00	1.10	0.38	1.85	386.48	388	0.39
14	25.00	1.20	0.31	1.99	402.03	442	9.04
15	24.00	1.20	0.28	2.10	270.76	256	5.77
16	25.00	1.10	0.27	2.39	317.34	335	5.27

*T*_tr_ true time measured by the survey

## Conclusions

This paper proposed a transfer time prediction model which was appropriate for different transfer stations. On the basis of the learning set, the model parameters of transfer speed and transfer volume were established with respect to the sectional number of transfer passengers. Through the testing set including 64 samples on four different transfer stations, the results demonstrated that the average error rate and the standard deviation were 3.50 % and 0.024. Results indicated that this transfer time model had the merits of accuracy, feasibility and reliability. By means of the model, transfer time could be predicted accurately. Furthermore, the proposal model was helpful for the operation department to coordinate subway trains between lines.

With more application of the model in the future, the regulation of transfer time should be further studied to analyze the distributions of waiting time and the transfer passage choice of passengers.
